# Topological polarization singular lasing with highly efficient radiation channel

**DOI:** 10.1038/s41467-022-34307-4

**Published:** 2022-10-30

**Authors:** Yun-Gang Sang, Jing-Yu Lu, Yun-Hao Ouyang, Hong-Yi Luan, Jia-Hao Wu, Jia-Yong Li, Ren-Min Ma

**Affiliations:** 1 State Key Lab for Mesoscopic Physics and School of Physics, Peking University, Beijing, China; 2 Frontiers Science Center for Nano-optoelectronic, Peking University, Beijing, China; 3 Peking University Yangtze Delta Institute of Optoelectronics, Nantong, Jiangsu China; 4 National Biomedical Imaging Center, Peking University, Beijing, China

**Keywords:** Nonlinear optics, Ultrafast lasers, Other photonics

## Abstract

Bound states in the continuum (BICs) in photonic crystals describe the originally leaky Bloch modes that can become bounded when their radiation fields carry topological polarization singularities. However, topological polarization singularities do not carry energy to far field, which limits radiation efficiencies of BICs for light emitting applications. Here, we demonstrate a topological polarization singular laser which has a topological polarization singular channel in the second Brillouin zone and a paired linearly polarized radiation channel in the first Brillouin zone. The presence of the singular channel enables the lasing mode with a higher quality factor than other modes for single mode lasing. In the meanwhile, the presence of the radiation channel secures the lasing mode with high radiation efficiency. The demonstrated topological polarization singular laser operates at room temperature with an external quantum efficiency exceeding 24%. Our work presents a new paradigm in eigenmode engineering for mode selection, exotic field manipulation and lasing.

## Abstract (web summary)

Here the authors develop topological polarization singular lasers that feature paired radiation channels carrying distinct topological properties which leads to single mode lasing with high external quantum efficiency.

## Introduction

Bloch modes can have distinct topological phases when their global geometric feature in a whole Brillouin zone is considered^1,2^. In 2008, Haldane and Raghu introduced band topology to the realm of photonics^3^. Since then, photonic analogues of varied topological phases including quantum Hall effect and quantum spin Hall effect have been intensively studied^4–12^. For photonics, the notion of topology provides a new powerful tool for field manipulation with robust feature. Compared to electronic systems, photonic systems are in general non-Hermitian, which gives rise to unique topological features^13–27^. Furthermore, structured lights propagating in free space can have topological features, such as phase and polarization singularities^28–32^. Recently, robust bound states in the continuum (BICs) have been connected to topological polarization singularities, where leaky Bloch modes can become bound states when their radiation fields carry topological polarization singularities^33–37^.

BICs have been widely introduced to laser physics and devices as a new tool of eigenmode engineering for robust, high dimension and low threshold lasing^38–44^. In a photonic crystal, BICs can localize light field with extremely high out-of-plane quality factor when the two lateral dimensions are substantially larger than resonant wavelength. Such a higher quality factor compared to other cavity modes can lead to single BIC mode lasing. However, the quality factor of the desired lasing radiation channel should be comparable to or lower than the quality factor of loss channels including material absorption and lateral leaky loss channels. This can be clearly seen from the external quantum efficiency of a laser that is proportional to $\frac{{Q}_{{{{{{\rm{loss}}}}}}}}{{Q}_{{{{{{\rm{loss}}}}}}}+{Q}_{{{{{{\rm{radiation}}}}}}}}$, where ${Q}_{{{{{{\rm{loss}}}}}}}$ and ${Q}_{{{{{{\rm{radiation}}}}}}}$ are quality factors of loss channels and lasing radiation channel respectively. The reported BICs lasers are with external quantum efficiencies of ~1% or even lower^43,44^. To achieve single mode lasing with high external quantum efficiency, a resonant control mechanism that can finely tune radiation field is required.

Here, we propose a cavity with two radiation channels with distinct topological features. Based on the proposed resonant mechanism, we demonstrate a topological polarization singular laser which has a topological polarization singular channel in the second Brillouin zone and a paired linearly polarized radiation channel in the first Brillouin zone. The presence of the singular channel enables the lasing mode with a higher quality factor than other modes for single mode lasing. In the meanwhile, the presence of the radiation channel secures the lasing mode with high radiation efficiency. The demonstrated topological polarization singular laser operates at room temperature with superior performance including low lasing threshold of ~1.0 kW/cm^2^, narrow linewidth of 0.14 nm, high external quantum efficiency of ~24.5% and high side mode suppression ratio of ~36.5 dB (Supplementary Section 1). The robust and movable features of topological polarization singularity in momentum space provide a controllable way to finely tune the quality factor, which opens new opportunity in achieving high performance laser devices at various scale.

## Results

### Polarization singularity in the second Brillouin zone

**Fig. 1:**
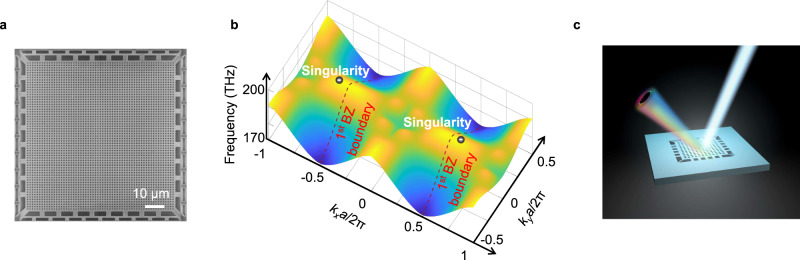
Scanning electron microscope (SEM) image and band diagram of a topological polarization singular laser. **a** SEM image of a topological polarization singular laser, where a semiconductor membrane serves as photonic crystal and gain material simultaneously. The lattice constant ($a$) is ~1240 nm, and the operation wavelength ($\lambda$) is ~1567 nm in free space. The length of the reciprocal lattice vector (${G}_{0}=\frac{2\pi }{a}$) is smaller than the diameter of light cone ($\frac{4\pi }{\lambda }$), which allows radiation to free space from the second Brillouin zone. **b** Three-dimensional full wave simulated band diagram of the device. Along ${k}_{x}$ direction, the radiation field from the second Brillouin zone supports a pair of polarization singularities with topological charge of −1. **c** Schematic of a topological polarization singular laser with paired singular (left emission beam) and radiation (right emission beam) channels. For clarity, only one pair of emission channels is shown.

The periodic structure of a photonic crystal introduces coupling between modes with momenta differed by reciprocal lattice vectors, which results in Bloch modes containing different components in various Brillouin zones. To reveal the distinct topological features of the radiation field of a Bloch mode in different Brillouin zones, we design our photonic crystal with a large lattice constant that allows not only the first Brillouin zone component to radiate inside the light cone but also the second Brillouin zone component. To do so, the length of the reciprocal lattice vector ${G}_{0}$ (${G}_{0}=\frac{2\pi }{a}$, $a$: lattice constant) needs to be smaller than the diameter of light cone ($2\times \frac{2\pi }{\lambda }$, $\lambda$: free space wavelength). Therefore, $a$ should be larger than $\frac{\lambda }{2}$. Figure 1a shows the scanning electron microscope (SEM) image of a fabricated photonic crystal with dual radiation channels, where a suspended 200 nm thick semiconductor membrane is etched with square-lattice nanoholes, serving as photonic crystal and gain material simultaneously (Methods). In the fabricated photonic crystal, the lattice constant is ~1240 nm, and the operation wavelength is ~1567 nm in free space, allowing radiation to free space from the second Brillouin zone.

Figure 1b shows the band diagram of the photonic crystal obtained by three-dimensional full wave simulation. To find topological polarization singularities in the radiation field of the structure, we use full simulation to obtain the radiation polarization vectors of all the Bloch modes inside the light cone (Methods and Supplementary Section 2). All these polarization vectors form a continuous vector field inside the light cone where any existing polarization singularity emerges. According to the simulation result, along ${k}_{x}$ or ${k}_{y}$ direction, the radiation field from the second Brillouin zone supports a pair of polarization singularities with topological charge of −1 (Supplementary Section 2). Each singular channel has a paired radiation channel with linear polarization in the first Brillouin zone.

Figure 1c shows the schematic of a topological polarization singular laser with a pair of singular and radiation channels. The momenta of singular and radiation channels are defined in the periodic structure of the photonic crystal. The in-plane momentum difference between a pair of singular and radiation channels is ${G}_{0}$. Their different in-plane momenta result in different out-of-plane radiation directions due to the conservation of momentum. The same principle can be applied to triangle lattice to construct a topological polarization singular laser cavity with a pair of singular and radiation channels (Supplementary Section 3).

### Topological polarization singular lasing

**Fig. 2:**
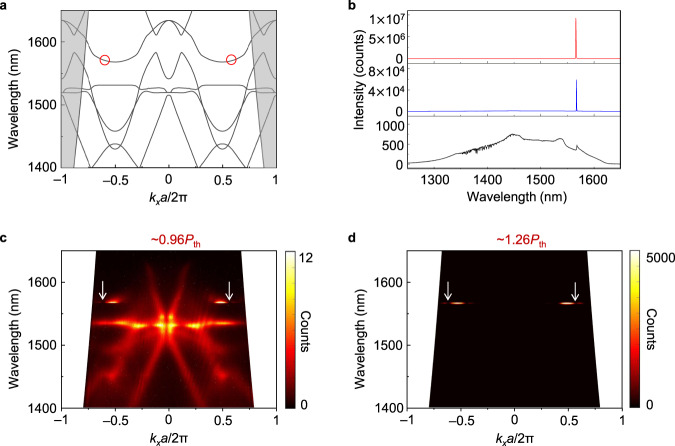
Topological polarization singular lasing with paired radiation channel. **a** Band diagram of the topological polarization singular lasing along Γ-*k*_*x*_ direction. The positions of two polarization singularities are highlighted by red circles in the second Brillouin zone. **b** Spontaneous emission and lasing emission of the device, which are pumped at ~0.96*P*_th_ (black), ~1.26*P*_th_ (blue), and ~5.70*P*_th_ (red) respectively. *P*_th_: lasing threshold. **c** Angle-resolved spontaneous spectra at ~0.96*P*_th_. The measured bands are broken at the positions of two polarization singularities indicated by arrows, because they do not carry energy to optical far field. **d** Angle-resolved lasing spectrum at ~1.26*P*_th_. The lasing emission strongly localizes around two singularities indicated by arrows in both spectral and momentum dimensions. The emission at the positions of two paired radiation channels are substantially stronger than the singular channels.

Figure 2a shows the band diagram of the fabricated structure along Γ-*k*_*x*_ direction, where the positions of two polarization singularities are highlighted by red circles in the second Brillouin zone. We first use spontaneous emission of the semiconductor membrane as a broadband probe to directly image the simulated band diagram and the polarization singularities on it (Methods). The black curve in Fig. 2b shows the spontaneous emission spectrum of the device when the device is pumped below lasing threshold at ~0.96*P*_th_ (*P*_th_: lasing threshold). Figure 2c shows the corresponding angle-resolved spectrum at the same pump power, where the simulated bands depicted in Fig. 2a are clearly observable. Remarkably, the measured bands are broken at the simulated positions of two polarization singularities along *k*_*x*_ direction, which clearly indicates the existence of the singularities because they do not carry energy to far field. The background emission in Fig. 2c should originate from the gain material radiation with dominant out-of-plane momentum that is not affected by periodic structure.

The device starts to lase above threshold (*P*_th_). At ~1.26*P*_th_, pronounced single-mode lasing appears as shown by the blue curve in Fig. 2b. Figure 2d shows the corresponding angle-resolved spectrum. In stark contrast to the spectrum below threshold, the emission now strongly localizes in both spectral and momentum dimensions. The single mode lasing is very stable with the increased pump power. At ~5.70*P*_th,_ the side mode suppression ratio reaches to ~36.5 dB (red curve in Fig. 2b, Supplementary Section 4). In spectral dimension, the emission localizes at ~1567 nm, which matches well with the wavelength of singularities in the band diagram (Fig. 2a). In momentum dimension, the emission localizes around the two singularities at $-1.082\frac{\pi }{a}$ and $1.082\frac{\pi }{a}$ respectively. The singularity at $-1.082\frac{\pi }{a}$ has the paired radiation channel at $0.918\frac{\pi }{a}$ ($=- 1.082\frac{\pi }{a}+{G}_{0}$), and the other singularity at $1.082\frac{\pi }{a}$ has the paired radiation channel at $-0.918\frac{\pi }{a}$ ($=1.082\frac{\pi }{a}-{G}_{0}$). Due to the finite cavity size, the emission beam in momentum dimension consists of a wave packet around the Brillouin zone boundary, which we use to visualize the paired radiation channels with distinct topological features. As shown in Fig. 2d, the emission at the positions of two paired radiation channels are substantially stronger than the singular channels. The coupled singular and radiation channel is the dominate loss channel in the system. According to full wave simulation, the radiation rate of the coupled channel is about an order of magnitude higher than the radiation rate of the in-plane leaky loss.

### Direct imaging polarization singularities

**Fig. 3:**
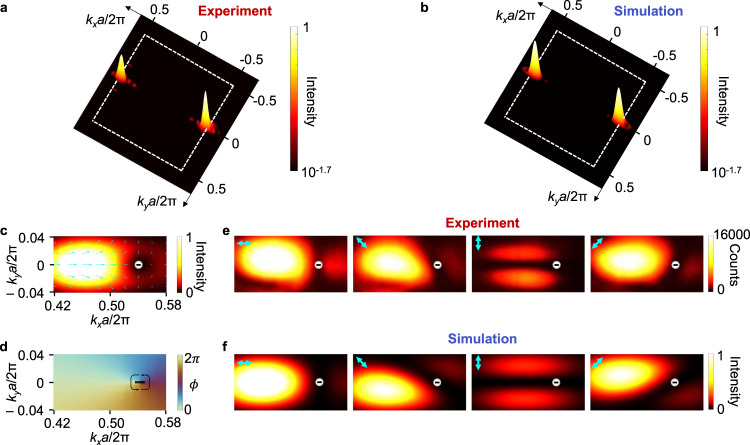
Direct imaging polarization singularities at lasing state. **a** Lasing emission pattern in momentum space. Two polarization singularities from the lasing mode appear as dark holes, and each of them has a paired radiation channel appearing as bright peak. **b** Three-dimensional full wave simulated lasing emission pattern in momentum space. The dashed squares in **a**, **b** indicate the boundary of the first Brillouin zone. **c** Simulated emission pattern superimposed with local polarization direction around a singularity and a radiation channel in momentum space. Arrows: polarization vectors indicating local electrical field polarization. **d** Direction of polarization vectors, where $\phi$ is the angle of polarization vectors with respect to positive *k*_*x*_-axis in counter-clockwise orientation. **e** Lasing patterns in momentum space after a linear polarizer with varied polarization angles. Arrows: directions of the linear polarizer. **f** Simulated emission patterns with the same polarization states as in panel **e**.

Because the emission linewidth is only ~0.14 nm above lasing threshold, the lasing emission can be used as a narrow band probe to characterize the equifrequency contour of the device radiation property around singular frequency. Figure 3a shows the momentum space image of the lasing emission, where the two polarization singularities appear as dark holes and their paired radiation channels appear as bright peaks. In the experiment, we only excite singularities along ${k}_{x}$ direction to simply the system, where the degeneracy between the modes along ${k}_{x}$ and ${k}_{y}$ axes are lifted by using a rectangular-shaped pump laser beam (Supplementary Section 5). Figure 3b shows the three-dimensional full wave simulated lasing emission distribution in momentum space, which matches well with the experimental data shown in Fig. 3a.

Figure 3c shows the simulated emission pattern superimposed with local polarization vectors around a singularity in momentum space. We can see that the singularity and the radiation channel have different polarization state. While the electric field is linearly polarized for the radiation channel, polarization vector rotates a full circle around the singularity. For a fixed rotating direction of observation, polarization vector rotates oppositely, indicating that the topological charge of the singularity is −1. Figure 3d shows the direction of polarization vectors only in the same area as in Fig. 3c, where the polarization singularity is clearly visualized.

To direct characterize the polarization states of the singular and radiation channels, we image the lasing pattern in momentum space after a linear polarizer with varied polarization angles. Figure 3e shows the imaged patterns at four typical angles, which clearly illustrate that the radiation channel is linearly polarized while the polarization vector rotates a circle around the singularity. Figure 3f shows the simulated polarized emission patterns, which match well with experimental ones.

### Lasing threshold behavior

**Fig. 4:**
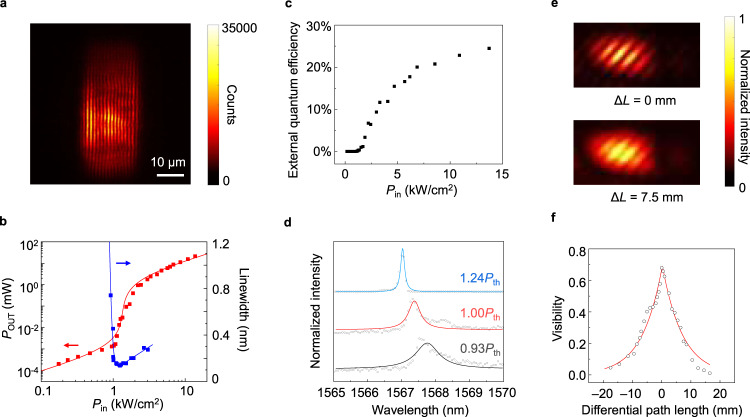
Threshold behavior in light-light and linewidth evolution curves. **a** Experimental emission patterns in real space. **b** Light-light curve of the device in log-log scale together with the linewidth evolution curve under varied pump power. **c** External quantum efficiency of the device under varied pump power. **d** Spectra around threshold. Circles: data; lines: fitting. **e** Interference patterns by superposing two split lasing emission beams at two different differential path lengths. Top: differential path length equals to 0. Bottom: differential path length equals to ~7.5 mm. **f** Visibility at varied differential path lengths, which is in Lorentz lineshape with a full-width at half maximum (FWHM) of 9.5 mm. Such a FWHM corresponds to a coherence time of 0.023 ns, and a spectral FWHM of ~0.11 nm, which matches with the one measured by spectrometer. Circles: data, line: fitting.

Figure 4a shows the lasing mission pattern in real space. In contrast to the spontaneous emission pattern (Supplementary Section 5), there is clear interference fringes at lasing state which is due to the interference between two counter propagating travelling Bloch modes along the *x* direction. The light-light curve and linewidth evolution curve are shown in Fig. 4b. The light-light curve is in S-shape, which indicates clearly the phase transition from spontaneous to stimulating emission. The spontaneous emission coupling factor is ~0.0006 by fitting the curve with rate equations (Supplementary Section 6). Laing threshold can be unambiguously defined at a pump power where the generated mean photon number inside the cavity mode is one^45–47^. Using such a quantum lasing threshold, we obtain the lasing threshold to be about 1.0 kW/cm^2^ from the curve. The high radiation rate of the radiation channel enables the topological polarization singular laser with high quantum efficiency exceeding 24% (Fig. 4c, Supplementary Section 7). Figure 4b also shows that the reduction of linewidth coincides with the threshold. The corresponding spectra around threshold are shown in Fig. 4d. The narrowest linewidth reaches to ~0.14 nm, corresponding to a lasing quality factor of 11,000.

To complement the linewidth analysis from measured lasing spectra, we also conduct the first-order coherence measurement shown in Fig. 4e, f. Figure 4e shows the interference patterns by superposing two splitted lasing emission beams at two different differential path lengths (Supplementary Section 8). When the differential path length equals to 0, we see clear interference fringes with a visibility of ~0.68. When the differential path length increases to 7.5 mm, the visibility decreases to ~0.21. Figure 4f shows the visibility at varied differential path lengths, which is in Lorentz lineshape with a full-width at half maximum (FWHM) of 9.5 mm. Such a FWHM corresponds to a coherence time of 0.023 ns, and a spectral FWHM of ~0.11 nm, which matches with the one measured by spectrometer.

The emission wavelength of the topological polarization singular laser can be tuned by varying the lattice constant and the diameter of the nanoholes. By adjusting these two parameters, we have demonstrated topological polarization singular lasers with lasing emission wavelength from 1313 nm to 1569 nm (Supplementary Section 9). The position of the polarization topological singularity in the momentum space can be robustly tuned by varying the ratio of the nanoholes diameter over lattice constant, which can be utilized to finely control the radiation property of a resonant mode (Supplementary Section 10). The lasing size of our device is scalable. We have demonstrated lasing at other three different sizes of 5.8 μm × 7.9 μm, 7.9 μm × 13.2 μm, 12.1 μm × 18.1 μm (Supplementary Section 11). The robust and high tunability of the topological polarization singular laser offers unique opportunities for realizing high performance lasers at various wavelengths and scales for practical applications.

## Discussion

A normal laser has one single desired lasing radiation channel where its quality factor is tuned as a whole. To achieve mode selection in single-channel laser, conventional paradigm focuses on increasing the contrast of quality factors of different modes to differentiate them in gain competition. However, with other parasitic loss channels, the quality factor of the lasing radiation channel is not the higher the better. Conventional paradigm on mode selection lacks the ability to finely tune the quality factor of the lasing radiation channel in a control manner.

In this work, we find that a Bloch band can have radiation field carrying topological charges from its second Brillouin zone but not the first one. The finding not only expands band topology to a partial component of one single Bloch mode but also provides an unconventional mode selection mechanism. The new mode selection mechanism bases on a laser with two radiation channels which are topological polarization singular channel and its paired linearly polarized radiation channel. Because topological polarization singularity does not carry energy to far field, the presence of the singular channel enables the lasing mode with a higher quality factor than other modes for single-mode lasing. The robust and movable features of topological polarization singularity in momentum space provide a controllable way to finely tune the quality factor. In the meanwhile, the presence of the radiation channel secures the lasing mode with high radiation efficiency. Future work can be conducted on synergistically optimizing cavity size, the order of the cavity mode, resonant wavelength and the position of the polarization singularity for high-performance lasing at various wavelengths and scales for practical applications.

## Methods

### Device fabrication

To fabricate topological polarization singular lasers, we use a nanostructured membrane of InGaAsP multiple quantum wells to serve as gain and photonic crystals simultaneously. The membrane consists of 6 well layers sandwiched in barrier layers and is capped by 10 nm InP. The well layers are In_x=0.56_Ga_1−x_As_y=0.938_P_1−y_ in 10 nm thickness. The barrier layers are In_x=0.734_Ga_1−x_As_y=0.57_P_1−y_. A 70 nm layer of SiO_2_ is deposited via plasma enhanced chemical vaper deposition to serve as a dry etch hard mask. We use electron beam lithography to define nanoholes in square-lattice with high resolution on the resist. Subsequently, the SiO_2_ hard mask is defined by inductively coupled plasma (ICP) etching. Another ICP dry etching process is used to etch through the 200 nm multi-quantum wells layer after removing the resist. Next, the SiO_2_ mask is removed by HF solution. Finally, to form a suspended membrane, we use HCl:H_2_O (3:1) to etch away the InP substrate (Supplementary Section 12).

### Full wave simulation

Cavity modes are simulated by finite-element method. We use the periodic structure to obtain the band structure, quality factor, and polarization pattern, and we use the structure with finite size to calculate field distributions and polarization of the lasing mode. The permittivity of the membrane is set to be 11.9. A perfect matched layer is added in the simulation to serve as boundary condition. To simulate rectangle pump laser spot, we add an imaginary relative permittivity of $-0.03$ and 0.26 inside and outside the pump area. The experimental observed modes are identified by comparing their field distributions in real and momentum spaces and polarization in momentum space.

### Optical characterization

The topological polarization singular lasers are pumped at room temperature by a pulsed laser at 1064 nm, where its pulse width and repetition rate are 5 ns and 12 kHz, respectively (Supplementary Section 13). To simplify the optical setup, an objective (100×, 0.82 numerical aperture) is used for the focus of excitation beam and collection of emission beam simultaneously. The collection parts need the high NA. The excitation part does not require the high NA, as the excitation beam is much larger than the free space wavelength in any direction. The signals are then guided to a near-infrared camera and a spectrometer for imaging and spectral analyzing. In order to obtain two polarization singularities along *k*_*x*_ direction, a rectangle mask is used in the pumping system to obtain a rectangle pumping spot (Supplementary Section 5). The absorptance of the semiconductor membrane with photonic crystal structure in the topological polarization singular laser is about 47% calculated from the simulation of the designed structure considering the material loss. The material loss is derived by measuring the transmittance and reflectance of the semiconductor membrane transferred on a transparent SiO_2_ substrate with a pump beam at 1064 nm. The external quantum efficiency is calculated by $\frac{{P}_{{{{{{\rm{OUT}}}}}}}/{hv}}{{P}_{{{{{{\rm{IN}}}}}}}/{{hv}}_{{{{{{\rm{IN}}}}}}}}$, where ${P}_{{{{{{\rm{IN}}}}}}}$ and ${{hv}}_{{{{{{\rm{IN}}}}}}}$ are pump power and pump photon energy respectively, and ${P}_{{{{{{\rm{OUT}}}}}}}$ and ${hv}$ are output power and emitted photon energy respectively. The resolution of our spectrometer is ~0.1 nm.

### Reporting Summary

Further information on research design is available in the Nature Research Reporting Summary linked to this article.

## Supplementary information

Supplementary Information

Lasing Reporting Summary

## Data Availability

We declare that the data supporting the findings of this study are available within the paper.
