# Supplementary material for: Topological polarization singular lasing with highly efficient radiation channel
Source: Nat Commun. 2022 Oct 30;13:6485. doi: 10.1038/s41467-022-34307-4 (PMC9617866; doi:10.1038/s41467-022-34307-4)
Supplement: Supplementary file 1 — Supplementary Information [file 41467_2022_34307_MOESM1_ESM.pdf]

Supplementary Information for

Topological polarization singular lasing with highly efficient radiation channel

Yun-Gang Sang[1,2ǂ], Jing-Yu Lu[1,2ǂ], Yun-Hao Ouyang[1,2ǂ], Hong-Yi Luan[1,2ǂ], Jia-Hao Wu[1,2], Jia-Yong Li[1,2], Ren-Min Ma[1,2,3,4*]

[1] State Key Lab for Mesoscopic Physics and School of Physics, Peking University, Beijing, China
[2] Frontiers Science Center for Nano-optoelectronics, Peking University, Beijing, China
[3] Peking University Yangtze Delta Institute of Optoelectronics, Nantong, Jiangsu, China
[4] National Biomedical Imaging Center, Peking University, China

ǂ These authors contributed equally to this work.
* Correspondence to: renminma@pku.edu.cn

Content

S1. Comparison of topological polarization singular laser with other small lasers 2

S2. Polarization singularities in the radiation field 3

S3. Topological polarization singular cavity in triangle lattice 5

S4. Side mode suppression ratio 6

S5. Pump beam and spontaneous emission pattern............7

S6. Spontaneous emission coupling factor............8

S7. External quantum efficiency 9

S8. First-order coherence characterization............10

S9. Topological polarization singular lasers with tunable lasing emission wavelength ...11

S10. Position of the topological charge at different structure parameters 12

S11. Topological polarization singular lasers with scalable lasing size 13

S12. Device fabrication............14

S13. Optical characterization 15

S1. Comparison of topological polarization singular laser with other small lasers

Table S1: Comparison of topological polarization singular laser with other small lasers

Reference	Type	Physical Size	Wavelength	Temperature	Threshold	λ/Δλ	EQE	SMSR	Directionality
**This work**	**TPL**	**~19 μm×49 μm**	**~1567 nm**	**RT**	**~1.0 kW/cm^2**	**~11000**	**24.5%**	**~36.5 dB**	**Yes**
Nature **541**, 196-199 (2017)	BIC laser	~19 μm×19 μm	1551.4 nm	RT	3.9 kW/cm^2	~4700	—	—	Yes
Nat. Commun. **12**, 4135 (2021)	BIC laser	~15 μm×15 μm	~1595 nm	RT	~1.5 kW/cm^2	~7300	~1.2%	—	Yes
arXiv: 1707.00181(2017)	BIC laser	—	~1560 nm	RT	—	—	—	—	Yes
Science **367**, 1018-1021 (2020)	BIC laser	—	552 nm	RT	~42 MW/cm^2	~5500	—	—	Yes
Nano Lett. **20**, 6005-6011 (2020)	BIC laser	50 μm×50 μm	647.7 nm	RT	~180 MW/cm^2	2590	—	—	Yes
Laser Photon. Rev. **15**, 2000411 (2021)	BIC laser	—	614 nm	RT	~100 kW/cm^2	~614	—	—	Yes
Nano. Lett. **21**, 9754-9760 (2021)	BIC laser	50 μm×50 μm	626 nm	RT	~11 kW/cm^2	~600	—	—	Yes
Nat. Photonics **15**, 758-764 (2021)	Fano BIC laser	>40 μm×12 μm	~1560 nm	RT	~10 kW/cm^2	~78000	~0.001%	—	Waveguide coupled
Proc. Natl. Acad. Sci. **110**, 13711-13716 (2013)	PhC laser	3 cm^2	~580 nm	RT	~1.8 kW/cm^2	—	~0.0003%	—	Yes
ACS Photonics **4**, 2117-2123 (2017)	PhC nanolaser (1D Nanobeam laser)	8 μm×0.58 μm	1556 nm	RT	~2.5 kW/cm^2	~390	—	—	Waveguide coupled
Nat. Commun. **4**, 2822 (2013)	PhC nanolaser (1D Nanobeam laser)	~4.6 μm×0.61 μm	1578 nm	RT	~0.18 kA/cm^2	~3220	~0.5%	—	No
Nat. Photonics **9**, 311-315 (2015)	PhC defect laser (Two coupled L3 cavity)	>5.1 μm×3.4 μm	~1540 nm	RT	~71 kW/cm^2	~4300	~0.2%	—	No
Appl. Phys. Lett. **104**, 121108 (2014)	PhC defect laser (H0 Defect array)	~120 μm×120 μm	1588 – 1601 nm	RT	~0.13 - 0.41 kW/cm^2	~8000	—	—	No
Opt. Express **13**, 8819 (2005)	PhC defect laser (Single cavity)	~4 μm×4 μm	1543 nm	RT	~2 kW/cm^2	~6700	—	—	No
Nano Lett. **17** (3), 1892-1898 (2017)	PhC defect laser (Single/double cavity)	>4 μm×3.1 μm	1491.3 nm	RT	~6.8 kW/cm^2	~2130	—	—	No
Nat. Commun. **6**, 8276 (2015)	PhC defect laser (L1-L3 cavity)	>1.4 μm×4.7 μm	1520 nm	RT	~0.02 kW/cm^2	~3800	—	—	No
Nat. Photonics **11**, 81-84 (2017)	PhC defect laser (H0 defect cavity)	>8.6 μm×4.6 μm	~1556 nm	RT	65 kW/cm^2	~2600	~0.004%	—	Waveguide coupled
Nat. Nanotech. **13**, 1042-1047 (2018)	BIC laser	~60 μm×108 μm	~825 nm	77 K	~70 MW/cm^2	2750	—	~30 dB	Yes
Science **284**, 1819-1821 (1999)	PhC defect laser (Dipole mode)	~5 μm×5 μm	1504 nm	143 K	~95 kW/cm^2	~7500	—	—	No

TPL: topological polarization singular laser; BIC: bound states in the continuum; PhC: photonic crystal; RT: room temperature
λ/Δλ: wavelength over linewidth; EQE: external quantum efficiency; SMSR: side-mode suppression ratio.

S2. Polarization singularities in the radiation field

In a two dimensional photonic crystal in x-y plane with lattice constant a in both x, y directions (Fig. S1a), according to Bloch theorem, electric field $\boldsymbol{E}(x,y,z)$ of all eigenmodes in the photonic crystal can be expressed in the form of Bloch wavefunction,

$$\boldsymbol{E}_{n,\boldsymbol{k}_\parallel}(x,y,z) = \boldsymbol{u}_{n,\boldsymbol{k}_\parallel}(x,y,z)e^{-i(k_x x + k_y y)}, \tag{1}$$

where $\boldsymbol{k}_\parallel = (k_x, k_y)$ is the in-plane component of the Bloch wavevector $\boldsymbol{k}$, $\boldsymbol{u}_{n,\boldsymbol{k}_\parallel}(x,y,z)$ is a periodic function with the same periodicity of the photonic crystal structure.

Further, the electric field of Bloch eigenmodes can be expended into Fourier series:

$$\boldsymbol{E}_{n,\boldsymbol{k}_\parallel}(x,y,z) = e^{-i(k_x x + k_y y)} \sum_{l_1,l_2} \boldsymbol{A}_{\boldsymbol{G}_{l_1,l_2}}(z) e^{-iG_0(l_1 x + l_2 y)}, \tag{2}$$

where $\boldsymbol{G}_{l_1,l_2}$ denotes reciprocal lattice vectors, $\boldsymbol{G}_{l_1,l_2} = G_0(l_1, l_2)$, $l_1, l_2 \in \mathbb{Z}$, $G_0 = 2\pi/a$, and

$$\boldsymbol{A}_{\boldsymbol{G}_{l_1,l_2}}(z) = \frac{1}{a^2} \iint_{\text{unit cell}} \boldsymbol{u}_{n,\boldsymbol{k}_\parallel}(x,y,z) e^{iG_0(l_1 x + l_2 y)} \mathrm{d}x\mathrm{d}y \tag{3}$$

is the Fourier coefficient of $\boldsymbol{u}_{n,\boldsymbol{k}_\parallel}$. We use h to denote the thickness of the photonic crystal slab, and $z = 0$ is set at the center. Every item on the right side of Eq. (2) will excite a plane wave with in-plane wavevector $\boldsymbol{k}_\parallel + \boldsymbol{G}_{l_1,l_2}$ in free space and we use $\boldsymbol{P}_{\boldsymbol{k}_\parallel + \boldsymbol{G}_{l_1,l_2}}$ to denote its polarization:

$$\boldsymbol{P}_{\boldsymbol{k}_\parallel + \boldsymbol{G}_{l_1,l_2}} = \boldsymbol{A}_{\boldsymbol{G}_{l_1,l_2}}(z \gg \lambda), \tag{4}$$

where λ is the wavelength of the Bloch eigenmode in the free space.

For the Bloch eigenmodes residing the light cone, each of them has a radiation field of plane wave. These radiation fields are generally elliptical polarized in *x-y* plane, and their dominant linear-polarization components form a continuous two dimensional vector field. The polarization singularities are the vortex centers in this vector field and can be characterized by the topological charges, the winding numbers of the polarization vectors. The topological charge q is defined as the number of the circles that the polarization vector rotates around the polarization singularity:

$$q = \frac{1}{2\pi} \oint_{C} \mathrm{d}\boldsymbol{k}_{\parallel} \cdot \nabla_{\boldsymbol{k}_{\parallel}} [\phi(\boldsymbol{k}_{\parallel})] . \tag{5}$$

$\phi(\boldsymbol{k}_{\parallel})$ is the angle of a polarization vector and C is a closed simple path in momentum space going around the polarization singularity counterclockwise.

To find polarization singularities in momentum space, we conduct three dimensional full wave simulation to obtain all the interested eigenmodes of our constructed photonic crystal, from which the corresponding polarization vectors of their radiation fields can be obtained in momentum space. In particular, we are interested in the topological polarization singularities of off-Γ point BIC modes. Fig. S1c shows the simulated polarization vector pattern in an area close to one boundary of the 1st Brillouin zone. There is a topological polarization singularity from an off-Γ point BIC mode in the area, which locates at the crossing of two nodal lines and is with a topological charge of -1.

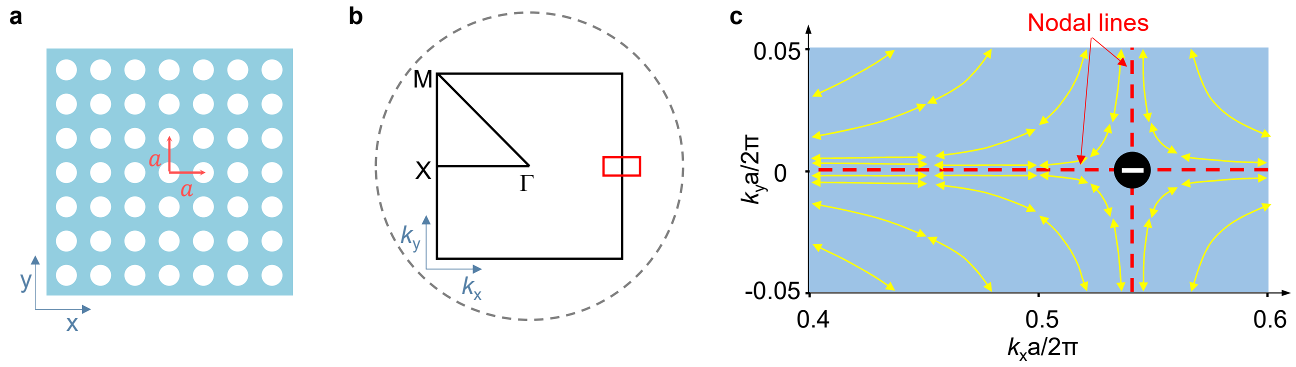

Fig. S1 | Polarization singularities in the radiation field. a, Schematic diagram in top view of our constructed photonic crystal where nanoholes in square-lattice are introduced into a semiconductor membrane. Red arrows are the two lattice vectors of the lattice. **b**, The first Brillouin zone and light cone of the lattice, which are represented by black square and dashed circle respectively. **c**, Simulated polarization vector pattern in the area indicated by the red rectangle in (**b**), which contains a topological polarization singularity with a topological charge of -1. Yellow arrows represent the directions of polarization vectors.

S3. Topological polarization singular cavity in triangle lattice

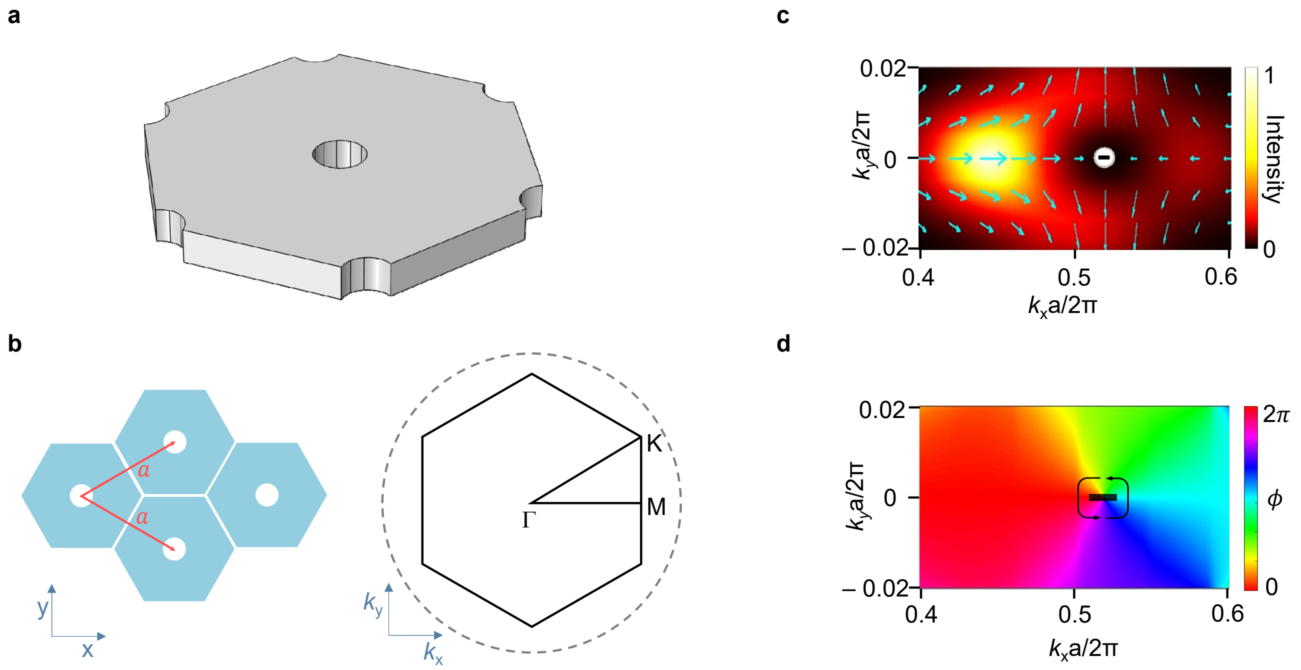

Fig. S2 | Topological polarization singular cavity in triangle lattice. a, Three-dimensional schematic of the full wave simulated triangle lattice. **b**, Top view of the lattice in real space (left) and the first Brillouin zone (right). Red arrows: lattice vectors of the lattice. The lattice constant is 1212 nm and the diameter of the nanoholes is 300 nm. **c**, Simulated emission pattern superimposed with local polarization direction around a singularity in the second Brillouin zone. **d**, Direction of polarization vectors in the same region as in (**c**), where ϕ is the angle of polarization vectors with respect to positive k_x-axis in CCW orientation.

S4. Side mode suppression ratio

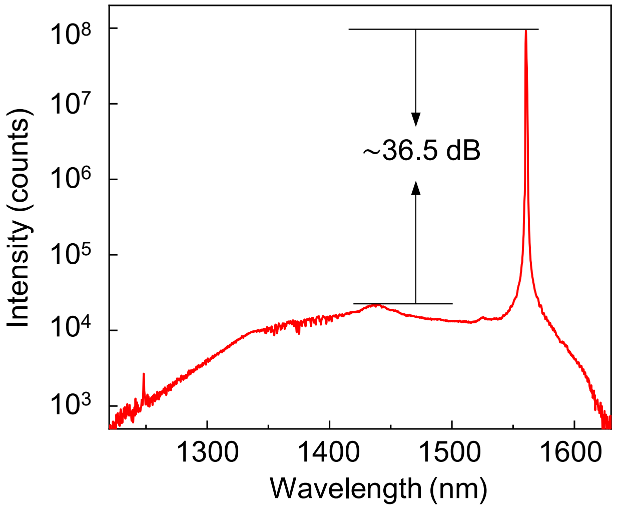

Fig. S3 | Lasing spectrum at a pump power density of ~5.7 kW/cm^2. The side mode suppression ratio is ~36.5 dB.

S5. Pump beam and spontaneous emission pattern

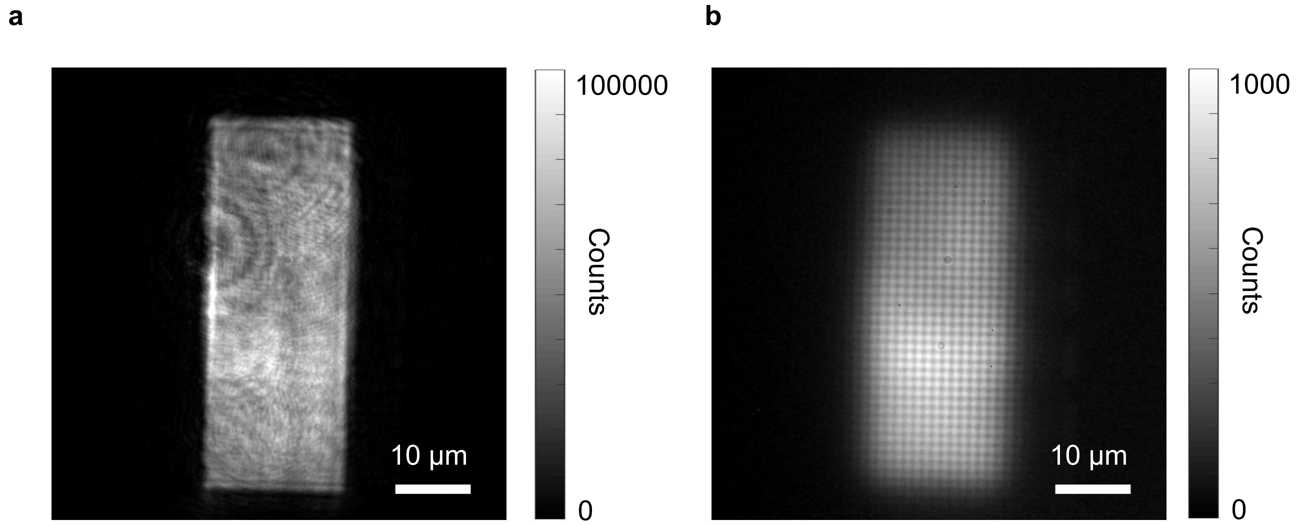

Fig. S4 | Pump beam at wavelength of 1064 nm and spontaneous emission in the real space. a, Pump beam at wavelength of 1064 nm. **b**, Spontaneous emission pattern in the real space.

S6. Spontaneous emission coupling factor

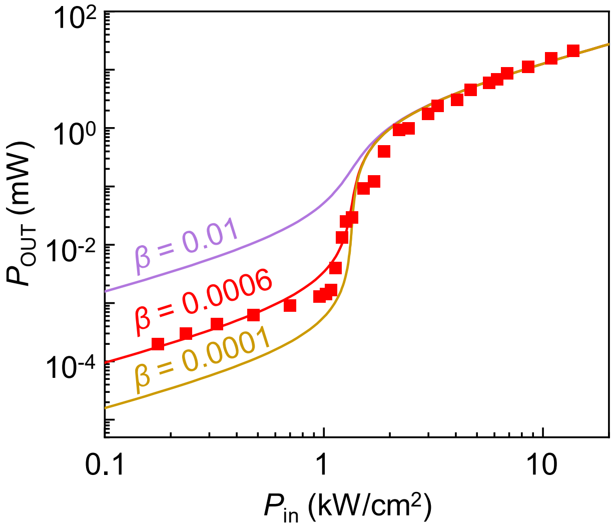

Fig. S5 | The light–light curve with fitting curves by rate equations. Red squares: experiment data. The spontaneous emission coupling factor β of the fitting curves are 0.01 (purple), 0.0006 (red) and 0.0001 (yellow), respectively.

S7. External quantum efficiency

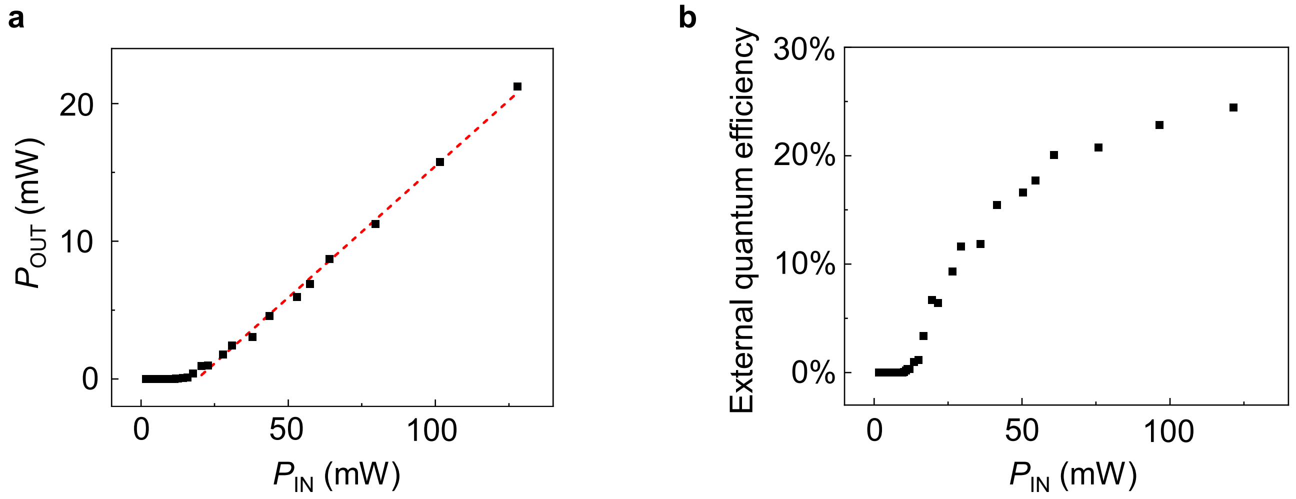

Fig. S6 | External quantum efficiency of the topological polarization singular laser. a, Light-light curve of the laser. P_{IN} and P_{OUT} are pump and output peak power respectively. **b**, External quantum efficiency of the laser under varied pump power.

S8. First-order coherence characterization

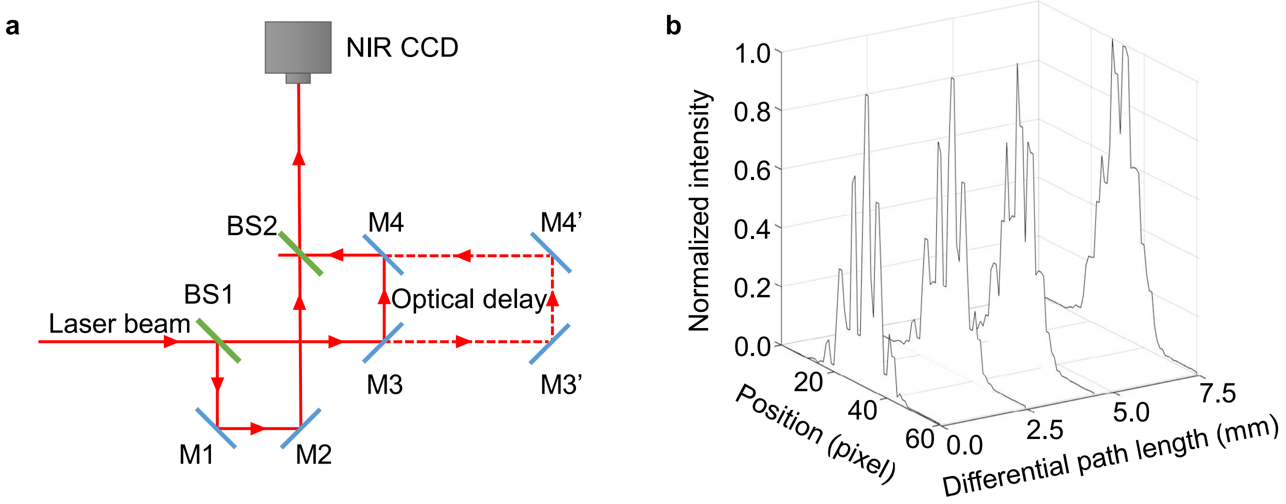

Fig. S7 | First-order coherence $g^{(1)}(\tau)$ characterization. a, The optical setup of the interferometer. Interference patterns are imaged by an infrared CCD camera. **b**, The cross sections of the interference patterns at varied differential path lengths (ΔL), where the decreased interference visibility with the increased ΔL is clearly observed.

S9. Topological polarization singular lasers with tunable lasing emission wavelength

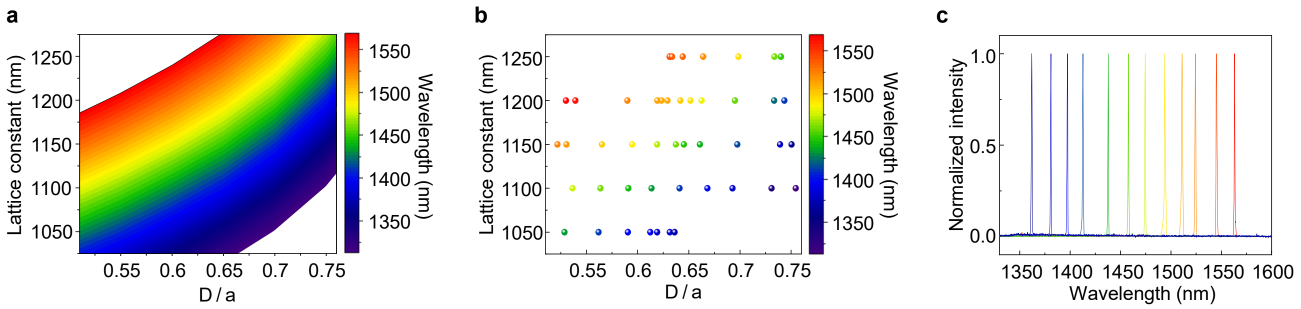

Fig. S8 | Topological polarization singular lasers with tunable lasing emission wavelength. a, **b**, Simulated cavity resonant wavelengths (**a**) and experimental lasing emission wavelengths (**b**) in the parameter spaces of lattice constant and the ratio of the diameter of nanoholes over the lattice constant (D/a). **c**, Typical lasing spectra in (**b**).

S10. Position of the topological charge at different structure parameters

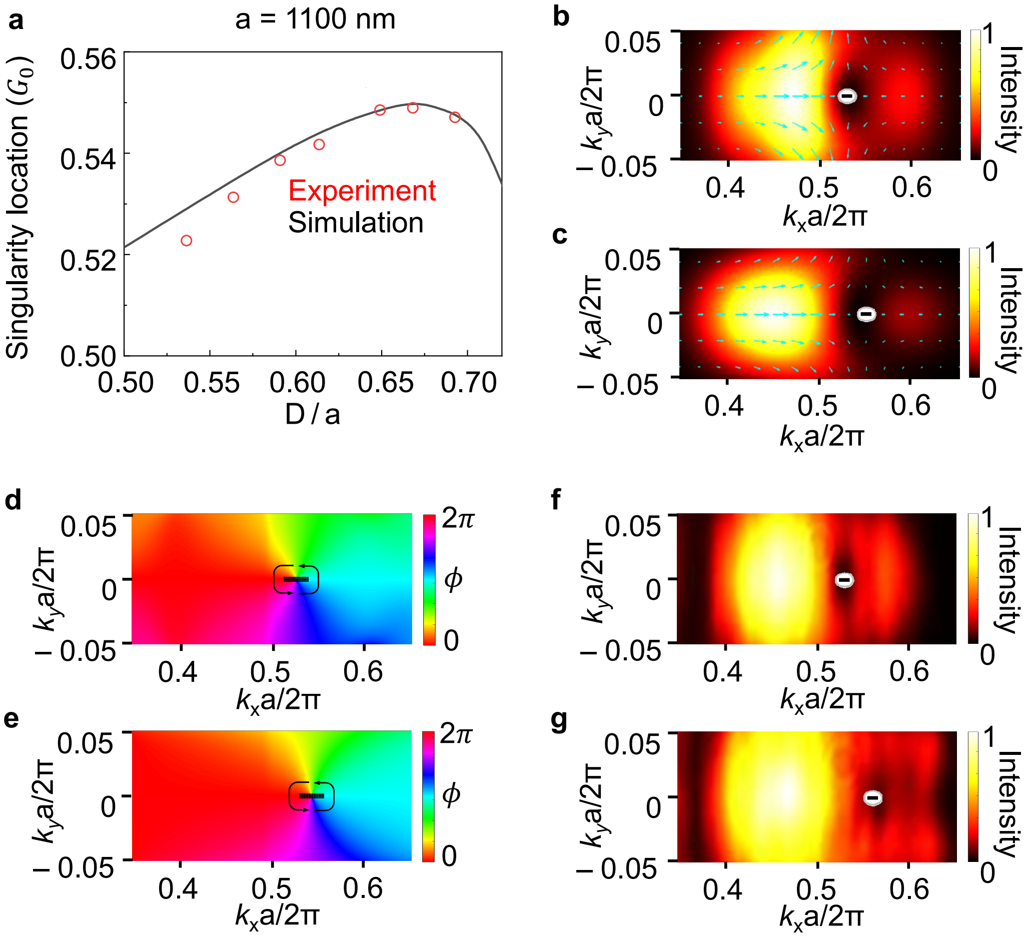

Fig. S9 | Position of the topological charge at different structure parameters. a, Position of the topological charge at varied ratio of diameter of nanoholes over lattice constant (D/a). The lattice constant is fixed at a = 1100 nm. Experimental and simulated results are represented by circles and line respectively. **b, c,** Simulated emission patterns superimposed with local polarization direction around a singularity at D/a = 0.536 (**b**) and D/a = 0.668 (**c**) respectively. Arrows represent polarization vectors indicating local electrical field polarization. **d, e,** Directions of polarization vectors at D/a = 0.536 (**d**) and D/a = 0.668 (**e**) respectively, where ϕ is the angle of polarization vectors with respect to positive k_x-axis in CCW orientation. **f, g,** Experimental obtained simulated emission patterns at D/a = 0.536 (**f**) and D/a = 0.668 (**g**) respectively, which match well with simulated ones shown in (**b**) and (**c**).

S11. Topological polarization singular lasers with scalable lasing size

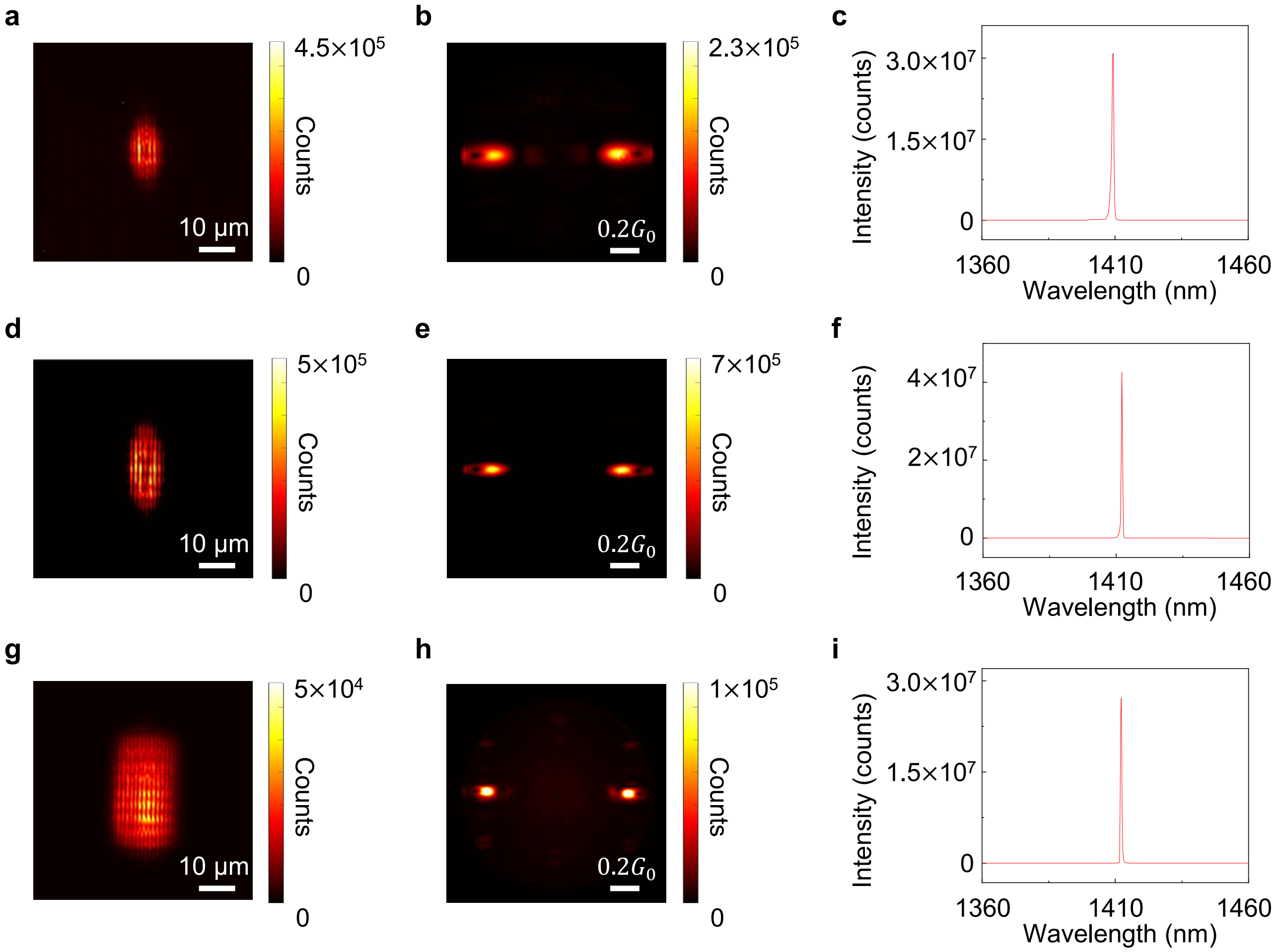

Fig. S10 | Topological polarization singular lasers with scalable lasing size. a-c, Lasing emission patterns in real space (**a**) and momentum space (**b**), and the corresponding spectrum (**c**) at the lasing size of ~5.8 μm×7.9 μm. **d-f**, Emission patterns in real space (**d**) and momentum space (**e**), and the corresponding spectrum (**f**) at the lasing size of ~7.9 μm×13.2 μm. **g-i**, Lasing emission patterns in real space (**g**) and momentum space (**h**), and the corresponding spectrum (**i**) at the lasing size of ~12.1 μm×18.1 μm.

S12. Device fabrication

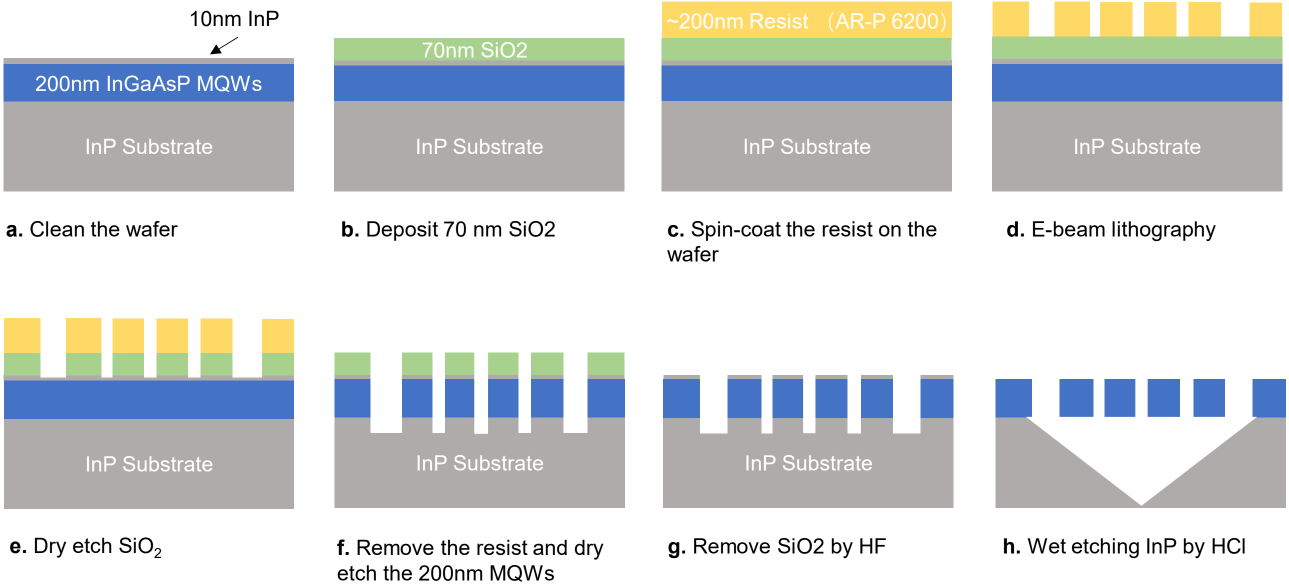

Fig. S11 | Flow chart of the fabrication. a, As-grown InGaAsP multiple quantum wells (MQWs) wafer. **b**, Deposition of a layer of SiO_2 hard mask with plasma enhanced chemical vaper deposition. **c**, Spin-coating E-beam resist of AR-P 6200 as mask. **d**, Creation of photonic crystal structure in the resist by E-beam lithography. **e**, Pattern transfer from the layer of resist to the layer of SiO_2 hard mask by dry etching. **f**, Removal of residual resist and pattern transfer from the layer of SiO_2 hard mask to the layer of MQWs by dry etching. **g**, Removal of SiO_2 hard mask by HF solution. **h**, Creation of suspended structure via wet etching of InP substrate.

S13. Optical characterization

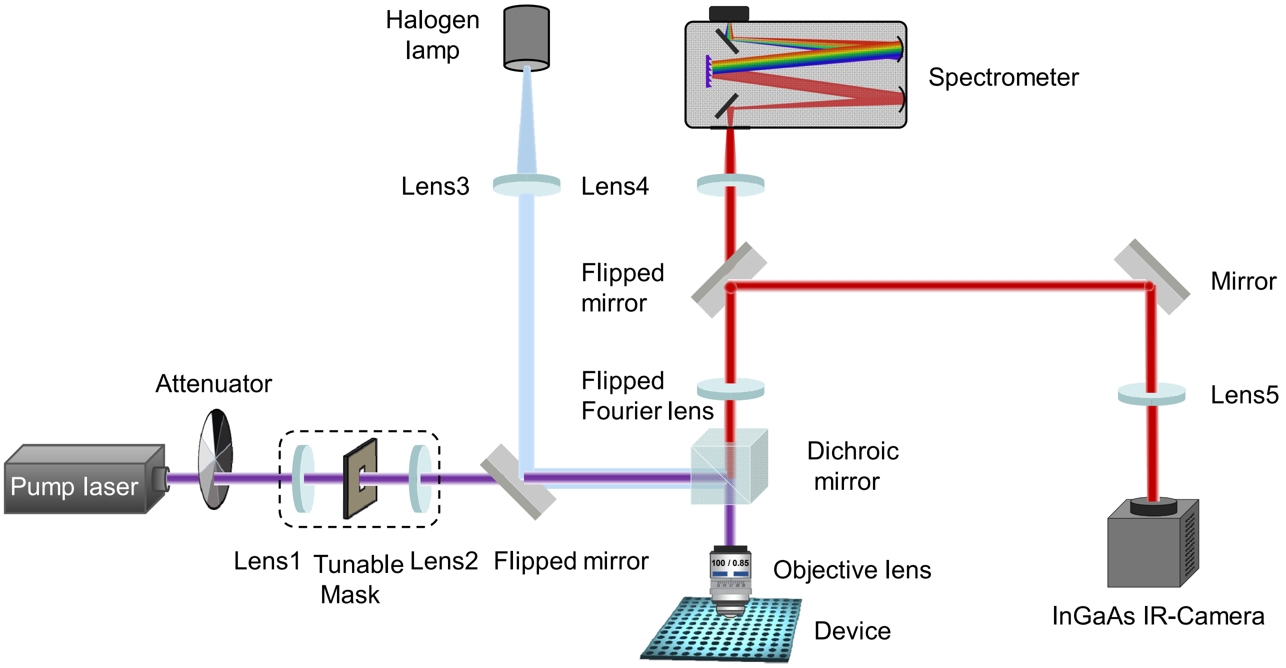

Fig. S12 | Experimental setup for lasing characterization. A nanosecond laser is used to pump the device (1064 nm, pulse length, 5 ns, repetition rate, 12 kHz). A microscope objective (×100) with a numerical aperture (NA) of 0.82 is used to focus the pump laser beam on the sample and collect the emission to an InGaAs camera and a near infrared spectrometer. The devices are pumped by imaging a rectangle mask onto the surface of the sample.
