# Supplementary material for: Topological polarization singular lasing with highly efficient radiation channel
Source: Nat Commun. 2022 Oct 30;13:6485. doi: 10.1038/s41467-022-34307-4 (PMC9617866; doi:10.1038/s41467-022-34307-4)
Supplement: Supplementary file 2 — Lasing Reporting Summary [file 41467_2022_34307_MOESM2_ESM.pdf]

nature research

Corresponding author(s): Ren-Min Ma

Lasing Reporting Summary

Nature Research wishes to improve the reproducibility of the work that we publish. This form is intended for publication with all accepted papers reporting claims of lasing and provides structure for consistency and transparency in reporting. Some list items might not apply to an individual manuscript, but all fields must be completed for clarity.

For further information on Nature Research policies, including our data availability policy, see Authors & Referees.

Experimental design

Please check: are the following details reported in the manuscript?

1. Threshold

Plots of device output power versus pump power over a wide range of values indicating a clear threshold

☒ Yes ☐ No

Fig. 4b in the main text, Fig. S6a in the supplementary information

2. Linewidth narrowing

Plots of spectral power density for the emission at pump powers below, around, and above the lasing threshold, indicating a clear linewidth narrowing at threshold

☒ Yes ☐ No

Fig. 4b and Fig. 4d in the main text

Resolution of the spectrometer used to make spectral measurements

☒ Yes ☐ No

Method section in the main text

3. Coherent emission

Measurements of the coherence and/or polarization of the emission

☒ Yes ☐ No

Fig. 3c in the main text

4. Beam spatial profile

Image and/or measurement of the spatial shape and profile of the emission, showing a well-defined beam above threshold

☒ Yes ☐ No

Fig. 3a and Fig. 4a in the main text, Fig. S10 in the supplementary information

5. Operating conditions

Description of the laser and pumping conditions
Continuous-wave, pulsed, temperature of operation

☒ Yes ☐ No

Method section in the main text

Threshold values provided as density values (e.g. W cm^{-2} or J cm^{-2}) taking into account the area of the device

☒ Yes ☐ No

Results section of the main text

6. Alternative explanations

Reasoning as to why alternative explanations have been ruled out as responsible for the emission characteristics
e.g. amplified spontaneous, directional scattering; modification of fluorescence spectrum by the cavity

☒ Yes ☐ No

We have systematically studied the lasing properties of our devices in experiment and in theory. These results are shown throughout the main text and the Supporting Information. The phase transition from spontaneous emission to lasing emission are verified by well defined lasing threshold, pattern, linewidth narrowing etc. which rules out other alternative explanations.

7. Theoretical analysis

Theoretical analysis that ensures that the experimental values measured are realistic and reasonable
e.g. laser threshold, linewidth, cavity gain-loss, efficiency

☒ Yes ☐ No

Fig. 2a, Fig. 3b, Fig. 3d in the main text; Fig. S5, Fig. S8 in the supplementary information

8. Statistics

Number of devices fabricated and tested

☒ Yes ☐ No

We have fabricated and tested over 100 lasing devices.

Statistical analysis of the device performance and lifetime (time to failure)

☒ Yes ☐ No

We have fabricated and tested over 100 lasing devices. Our devices are based on III-V semiconductor, they did not show performance degradation during the test period of over two years.
